# Use of physical restraints on older adults in South Korean nursing homes: a multicenter study

**DOI:** 10.1038/s41598-023-50897-5

**Published:** 2024-01-03

**Authors:** Eunhee Cho, Deulle Min

**Affiliations:** 1 Yonsei University College of Nursing & Mo-Im Kim Nursing Research Institute, Seoul, Republic of Korea; 2 Department of Nursing, College of Medicine, Wonkwang University, 460, Iksandae-ro, Iksan, Jeonbuk 54538 Republic of Korea

**Keywords:** Health care, Risk factors

## Abstract

In this study, we aimed to examine the current status of physical restraint use and ascertain factors affecting the rate of usage of physical restraints on older adults in South Korean nursing homes. For this purpose, we conducted a secondary analysis of data from 190 registered nurses employed at 62 nursing homes. Logistic regression analysis was used to identify the factors affecting the use of restraints in nursing homes. The rate of using physical restraints was 79.5%. Nursing homes were found to use 90.7% and 91.3% less restraint when the work environment was better (odds ratio [OR]: 0.093, 95% confidence interval [CI]: 0.023–0.368) and mixed (OR: 0.087, 95% CI: 0.087–0.100), respectively. Nursing homes owned by corporations were 9.796 times more likely to use physical restraints than those owned by local governments (OR: 9.796, 95% CI: 1.473–65.158). Therefore, improving nurses’ work environment and introducing regulations and education that enable the entrusted doctors or nurses to make decisions regarding restraint use, monitoring, and removal, regardless of nursing home ownership type, are necessary.

## Introduction

Over the centuries, “physical restraint” has been defined in various ways, but in 2011, the Annual Scientific Meeting of the Gerontological Society of America, an international group of 47 experts from 14 countries, came to the following consensus: “Physical restraint is defined as any action or procedure that prevents a person’s free body movement to a position of choice and/or normal access to their body by the use of any method, attached or adjacent to a person’s body they cannot control or remove easily”^1^. In many cases, the use of restraints is aimed at protecting the person with physical or cognitive impairments from injuries due to falls, safeguarding the therapeutic equipment, and shielding others from acts of aggression. Types of restraints include bedsheets, safety vests, belt restraints, limb restraints, and special chairs for older adults^2,3^.

However, previous studies have pointed out that there are cautionary issues in applying physical restraint. Some have reported that people who are physically restrained are more likely to have decreased cognitive functions, limitations in activities of daily living and walking ability, and a higher frequency of falls. Restraint use may also lead to psychological complications such as depression, agitation, and anxiety and physical complications such as pressure ulcer, urinary incontinence, constipation, deep vein thrombosis, aspiration pneumonia, and even death^3–5^. In addition, geriatric or critical care nurses who apply the physical restraints may feel guilty as they face ethical dilemmas related to violating the principle of good deeds that guides the profession^5,6^.

Restraint use is known to be influenced by patient and resident characteristics, such as age and physical and cognitive function; nurse characteristics, such as knowledge and attitudes toward restraint use; and organizational characteristics, such as staffing, work environment, and working conditions^3,5,7–12^. Regarding nurses’ working conditions, inadequate coworker support, high job demands, and low control have been reported to be closely related to the use of restraints^11,12^. These factors have led to the continued use of physical restraint in nursing homes in many countries. The prevalence of physical restraint use in Western nursing homes varies from 6 to 65%^13–16^. In the Asian context, a study examining the prevalence of restraint use in 14 nursing homes in Hong Kong reported a rate of 20%^16^. In South Korea, while the use rate of physical restraints in acute hospitals is reported to be about 30%^17–19^, there are few studies that can identify the prevalence of physical restraints in the country’s nursing homes.

South Korea’s Long-term Care Insurance Act mandates one registered nurse (RN) or certified nursing assistant (CNA) per 25 beds in its nursing homes. However, since this regulation allows CNAs to replace RNs and nursing homes prefer hiring CNAs to reduce their labor costs, Korean nursing homes now face a shortage of RNs^20,21^. This problem is further exacerbated by the fact that South Korea is rapidly heading toward a super-aged society, with demands for nursing homes growing accordingly^22^. However, as Korean nursing homes do not have resident doctors or advanced practice registered nurses, and about 60% of them do not even have RNs, use of restraints is not based on assessment and decision-making by healthcare professionals^23^. Furthermore, it is reported that RNs’ retention intention in nursing homes is not high owing to the poor work environment^24^. Nevertheless, few studies have investigated the association between nurses’ work environment and physical restraint use in South Korean nursing homes. Therefore, we aimed to: (1) examine the current status of physical restraint use in nursing homes in South Korea and (2) explore the various factors that affect the use of physical restraints in South Korean nursing homes. The results of this study can form the foundation for future practices and contribute to the provision of quality nursing care and policies for older adults.

## Material and methods

### Study design and participants

This is a secondary data analysis of a prior study on the development of tools to assess the working environment of nurses in South Korean nursing homes^25^. In the original study^25^, the inclusion criterion was RNs who were providing direct care at nursing homes with more than 100 beds in six metropolitan cities, Gyeonggi province, and Seoul. Gyeonggi province is larger than a typical metropolitan city, while Seoul qualifies as a megacity because it exceeds the 10 million population threshold, the conventional criterion for megacity classification^26^. The exclusion criterion was being the owner of a nursing home.

We used information posted on the long-term care facility website to identify nursing homes with RNs (https://longtermcare.or.kr). There were a total of 1588 nursing homes in Seoul, Gyeonggi province, and the six metropolitan cities, of which 1122 with no RNs were excluded, leaving 466 nursing homes that met the inclusion criterion. Of these, 107 nursing homes with more than 100 beds were invited to participate in the study, and only 64 agreed to participate. Researchers distributed questionnaires to all 224 RNs working at the 64 nursing homes and collected voluntarily completed questionnaires from 219 RNs affiliated with 62 nursing homes. Subsequently, we analyzed the data of 190 RNs who had answered all items related to restraint use. This number was found to have a power of about 93% at an alpha level of 0.05 and an odds ratio (OR) of 1.9, in line with a study investigating factors affecting the use of restraints in a nursing home in Switzerland using logistic regression^27^.

### Variables

#### Use of physical restraints

Regarding the use of physical restraints, RNs in nursing homes were surveyed on a 6-point Likert scale (0 = no, 1 = several times a year, 2 = once a month, 3 = several times a month, 4 = once a week, 5 = several times a week, and 6 = daily). This was reconstructed as 0 = no and 1–6 = yes for the final analysis model. In addition, if the answer was “yes,” whether restraints were used every day (yes/no) and whether they were used for more than eight hours every day (yes/no) were classified in detail.

#### Characteristics of nursing homes

All variables were derived from previous studies that reported nurses’ work environment and the institutional characteristics of nursing homes^3,9,10,25^. Number of beds (100–199 or ≥ 200), nurses’ work environment (poor, mixed, and better), ownership of nursing home (corporation, local government, and private), region (Seoul, metropolitan, and province), and number of RNs and CNAs in nursing homes were investigated. Among them, the nurses’ work environment was measured using 27 items divided into five factors (nurses’ participation in nursing home affairs; well-defined scope of practice; nurse managers’ ability, leadership, and support of nurses; staff and resource adequacy; and communication and coordination). Each item was rated on a 4-point Likert scale ranging from 1 (not at all) to 4 (strongly agree). The domain values calculated for each individual were aggregated at the institutional level. The average and median values of each domain were changed to 0 if the average value of each nursing home was less than the median value of 62 nursing homes, and to 1 if the average value was greater than or equal to the median value. These aggregates were grouped into three categories denoting the work environment of each nursing home: 0–1 for poor, 2–3 for mixed, and 4–5 for better. The reliability at the time of development of each factor was Cronbach’s alpha = 0.77–0.88^25^.

#### Characteristics of RNs

Based on previous studies, we investigated the characteristics of RNs according to the characteristics of nursing homes^24,25^. In addition, the number of patients the nurse took care of during their last shift, working period as a nurse (years), education (diploma vs. baccalaureate), and employment type (permanent vs. temporary) were examined.

### Data analysis

The collected data were analyzed using PASW SPSS WIN 23.0 (IBM Corp, Armonk, NY, USA). The characteristics of nursing homes and nurses were analyzed using descriptive statistics (percentage and average). Chi-square analysis was used for the use of physical restraints according to nursing home characteristics. Logistic regression analysis, including all characteristics of nursing homes and RNs, was used to identify factors influencing restraint use in nursing homes. In the final logistic regression analysis, the variable of the number of patients was eliminated owing to the number of beds, number of RNs, and risk of multi-correlations (r = 0.46, p < 0.001; r = 0.32, p < 0.001, respectively).

### Ethical considerations

This study was conducted after obtaining approval from the Institutional Review Board of the first author’s institution (no. Y-2015-0024-8). All participants provided written informed consent.

### Ethics approval and consent to participate

This study was conducted after obtaining approval from the Yonsei University Health System Research Ethics Committee (no. Y-2015-0024-8). All participants provided written informed consent. All methods were carried out in accordance with relevant guidelines and regulations.

## Results

### Characteristics of nursing homes and RNs

Of the 62 nursing homes, 82.3% had 100–199 beds. The nursing work environment was classified as follows: 27.4% poor, 41.9% mixed, and 30.6% better. Most nursing homes were operated by corporations (71.0%), and most were located in metropolitans (38.7%) and provinces (32.3%). The average number of RNs at each facility was approximately six and there were approximately three CNAs. Of the 190 RNs, 64.2% worked in nursing homes with 100–199 beds. The number of RNs who worked in places where the working environment was better or mixed was 52.1%. Most of them worked for a corporation (56.8%), and were from Seoul (41.6%). The average number of older adults the RNs were in charge of during their last duty was 78.67 ± 54.01, and the average work experience was 15.05 ± 8.4 years. Most RNs had graduated with a diploma (57.4%) and were permanent employees (89.5%) as shown in Table 1.

**Table 1:** Characteristics of nursing homes and registered nurses.

Variables	Category	NH (N = 62)	RN (N = 190)	Range
		n (%)/M ± SD	n (%)/M ± SD	
Number of beds	100–199	51 (82.3)	122 (64.2)	
	≥ 200–299	11 (17.7)	68 (35.8)	
Work environment	Poor	17 (27.4)	91 (47.9)	
	Mixed	26 (41.9)	53 (27.9)	
	Better	19 (30.6)	46 (24.2)	
Ownership	Corporation	44 (71.0)	108 (56.8)	
	Local government	10 (16.1)	69 (36.3)	
	Private	8 (29.0)	13 (6.8)	
Region	Seoul	18 (29.0)	79 (41.6)	
	Metropolitan	24 (38.7)	46 (25.8)	
	Gyeonggi province	20 (32.3)	62 (32.6)	
Number of RNs		5.89 ± 3.86		1–16
Number of CNAs		2.88 ± 2.35		0–11
Number of patients		–	78.67 ± 54.01	10.00–280.00
Working period (Years)		–	15.05 ± 8.40	0.58–40.75
Education	Diploma	–	109 (57.4)	
	Baccalaureate	–	81 (42.6)	
Employment type	Permanent	–	170 (89.5)	
	Temporary	–	20 (10.5)	

Note: NH, nursing homes; RN, registered nurse; CNA, certified nursing assistant.

### Use of physical restraints in nursing homes

The rate of usage of restraints in nursing homes was 79.5%, and 21.2% of the RNs reported usage of restraints every day. In 20.5% of cases, restraints were used for more than 8 h daily as shown in Table 2.

**Table 2:** Use of physical restraints in nursing homes (N = 190).

Variables	Category	Options	n (%)
Restraint use	No		39 (20.5)
	Yes		151 (79.5)
Details of “Yes” to restraint use (N = 151)			
	Everyday use	Yes	32 (21.2)
		No	119 (78.8)
	Use for more than 8 h every day	Yes	31 (20.5)
	(Missing = 6)	No	114 (75.5)

Note: n, number of participants.

### Use of physical restraints according to characteristics of nursing homes

Table 3 shows the differences in the use of physical restraints according to the characteristics of nursing homes. Nursing homes with 100–199 beds were 19.2% more likely to use physical restraints than nursing homes with 200 or more beds (*X*^2^ = 5.86, p = 0.016). Nursing homes with a poor work environment used physical restraint about 30% more than those with mixed or better environments (*X*^2^ = 8.85, p = 0.012). In addition, the use rate of physical restraints was higher when the ownership was by a corporation (*X*^2^ = 6.98, p = 0.030) and in nursing homes with a higher number of CNAs (*X*^2^ = 7.91, p = 0.005). There was no specific difference in the use rate of restraints based on region and number of RNs (Table 3).

**Table 3:** Use of physical restraints according to characteristics of nursing homes.

Variables	Category	Restraint use (Yes = 151)	
		n (%)	*X*^2^ (p)
Number of beds	100–199	90 (59.6)	5.86 (0.016)
	≥ 200	61 (40.4)	
Work environment	Poor	84 (55.6)	8.85 (0.012)
	Mixed	33 (21.9)	
	Better	34 (22.5)	
Ownership	Corporation	91 (60.3)	6.98 (0.030)
	Local government	48 (31.8)	
	Private	12 (7.9)	
Region	Seoul	60 (39.7)	1.18 (0.553)
	Metropolitan	50 (33.1)	
	Gyeonggi province	41 (27.2)	
Number of RNs	1–3 (25% quartile)	36 (23.8)	5.49 (0.140)
	4–6 (50% quartile)	45 (29.8)	
	7–9 (75% quartile)	38 (25.2)	
	10–16 (100% quartile)	32 (21.2)	
Number of CNAs	≤ 2 ≥ 3	64 (42.4) 87 (57.6)	7.91 (0.005)

Note: RN, registered nurse; CNA, certified nursing assistant.

### Factors affecting the use of physical restraints

The number of RNs and CNAs was not a significant factor in the use of physical restraints in nursing homes. Nursing homes were found to use 90.7% less restraint when the work environment was better (OR: 0.093, 95% confidence interval [CI]: 0.023–0.368) and 91.3% less restraint when the work environment was mixed (OR: 0.087, 95% CI: 0.087–0.100). When the ownership was by a corporation, physical restraint was used 9.796 times more (OR: 9.796, 95% CI: 1.473–65.158) than when the ownership was by a local government as shown in Table 4.

**Table 4:** Factors affecting the use of physical restraints.

Variable	Category	OR	95% CI	p-value
Number of beds (Ref: 100–199)	≥ 200–299	2.672	0.395–18.074	0.313
Work environment (Ref: Poor)	Mixed	0.087	0.087–0.100	0.033
	Better	0.093	0.023–0.368	0.001
Ownership (Ref: Local government)	Corporation	9.796	1.473–65.158	0.018
	Private	13.061	0.907–188.039	0.059
Region (Ref: Seoul)	Metropolitan	0.224	0.035–1.439	0.115
	Gyeonggi province	1.365	0.324–5.747	0.671
Number of RNs		1.037	0.804–1.337	0.780
Number of CNAs		1.574	1.028–2.412	0.759
Working period (Years)		1.034	0.968–1.106	0.318
Education (Ref: Diploma)	Baccalaureate	0.404	0.129–1.261	0.119
Employment type (Ref: Temporary)	Permanent	3.270	0.527–20.300	0.203

Note: OR, odds ratio; CI, confidence interval; RN, registered nurse; CNA, certified nursing assistant.

## Discussion

This study was conducted to understand the current status of physical restraint use in nursing homes in South Korea. The results show that 79.5% of nursing homes used physical restraints. This is higher than in studies on domestic hospitals showing 30–40% usage rates^17–19^. However, as there has been no research on the use of physical restraints in South Korean nursing homes, it is difficult to compare the results of this study.

The results showed that the factor affecting the use of restraints in nursing homes was nurses’ work environment; there was less use of restraints when the work environment was better. This is consistent with previous studies showing that nurses are more likely to use restraints in work environments with less autonomy^13^ and lack of support from managers and coworkers^10^. On the contrary, our results did not show the number of RNs to be a factor influencing the use of restraints. This is similar to the results of previous studies showing the lack of a relationship between the use of restraints and the level of staffing^12,13,28–30^. A study that examined the relationship between staffing and the use of restraints indicated that less than 1% of the participating nurses felt that they were using restraints owing to a lack of staffing^31^.

However, staff in nursing homes reported that organizational culture is a major barrier to reducing restraint use. The organizational culture needs to be changed to be supportive of reducing restraint use, fostering open discussion, and facilitating better collaboration among nurses, residents, families, doctors, and other staff members^32,33^. Staff education plays a crucial role in facilitating the desired transformation of organizational culture. Previous studies have reported that staff education is an important factor in reducing restraint use. With regard to this, staff members in nursing homes have been known to reveal a perceived need for education encompassing various aspects, including the definition of restraints, legal considerations, and, most notably, alternative approaches to restraint use^32–34^.

Another factor affecting the use of physical restraints identified was the ownership of nursing homes. Our results showed that nursing homes operated by corporations used more physical restraint compared to local government ownership. Private nursing homes also showed a higher frequency of utilizing physical restraints when compared to nursing homes owned by the local government; however, this difference was not statistically significant. Previous studies related to ownership have reported higher use of restraints in for-profit nursing homes^35,36^; however, one study reported that ownership is not related to physical restraint use^25^. Further research is required to investigate the specific characteristics of nursing homes based on ownership that contribute to a higher usage of physical restraints.

The existing body of literature lacks research data regarding the decision-making process for physical restraint use in South Korean nursing homes, as well as the responsible parties, including those who make the decision, apply the restraints, and monitor restraint usage. Based on the Medical Institute Evaluation in South Korea, acute hospitals have guidelines for continuous observation and evaluation after applying physical restraint to the patient. In 2013, Enforcement Regulations of the Medical Law for the application of physical restraints were established for long-term care hospitals^37^. This law requires that a doctor’s order be obtained before using physical restraints, including consent from the patient or family. In addition, when physical restraints are applied in long-term care hospitals, the patient is periodically observed and recorded to prevent side effects^37^. However, these regulations for restraint use do not apply to Korean nursing homes.

The use of restraints is an important factor affecting the safety and quality of life of older adults in nursing homes. Studies conducted in Germany and Spain show that various interventions in nursing homes, based on guidelines, have reduced the use of restraints^38,39^. The Physical Restraints Critical Element Pathway by the Centers for Medicare & Medicaid Services specifies that physical restraints should be used minimally. It cautions against their use for the convenience of employees or at the request of family members. Further, it emphasizes the necessity of an active care plan aimed at reducing the use of physical restraints^40,41^. To enhance the safety and overall well-being of older adults, the establishment of protocols and regulations is imperative. These regulations should require qualified healthcare professionals, such as doctors or nurses possessing the necessary expertise, to assess older adults’ condition to make decisions regarding the implementation, monitoring, and removal of physical restraints. The decision-making process for utilizing physical restraints should be based on a comprehensive and individualized assessment of the residents’ condition, which takes into account various factors including their medical needs, behavior patterns, and the complications and adverse effects associated with restraint use^38–41^. However, Korean nursing homes have no resident doctors or advanced practice registered nurses, and many do not have RNs either. Therefore, it is important not only to establish regulations and guidelines governing the use of physical restraints within nursing homes but also to create regulations mandating the presence of RNs in Korean nursing homes. This will ensure that qualified healthcare professionals oversee the implementation of and adherence to these regulations regarding the use of physical restraints. Mandatory placement of RNs in nursing homes would enable comprehensive assessments of residents’ conditions; person-centered decisions regarding the application, monitoring, and removal of physical restraints; as well as the minimal use of physical restraints. Their presence would also facilitate continuous observation and evaluation after applying physical restraints to prevent complications and adverse effects associated with physical restraints.

This study has the following limitations. First, it is a secondary data analysis; it did not include the age, physical and cognitive function status, and health-related information of older adults, which are known to be a common cause of the use of physical restraints. As reported in previous studies^3,5,7,8^, the health status of the older adults or patients is an important factor in the use of physical restraints; therefore, future studies should include them. Second, as the original study was carried out in nursing homes with RNs, the rate of use of restraints in nursing homes without RNs is unknown. Furthermore, we did not reveal the characteristics of RNs who did not participate in the study. Therefore, caution should be exercised when attempting to generalize the results, and studies should be conducted to examine restraint use and related factors in nursing homes without RNs as well as the characteristics of RNs who did not participate in this study. Third, owing to the use of self-reports, the possibility of recall bias cannot be ignored. In addition, it is difficult to ascertain the type of physical restraints the participants used. In future studies, it is necessary to include methods such as chart review to confirm the clear numerical value of restraint use including the types of physical restraints.

Despite these limitations, this study can form the basis for inquiry into nationwide physical restraint use rate in nursing homes, which has not been done before. The study’s results can serve as the foundation for future policies on the application of physical restraint to improve the safety and quality of life of older adults.

## Conclusions

Ownership of the nursing home and nurses’ work environment, rather than the number of RNs employed, were found to influence the use of physical restraints on older adults in nursing homes. Based on the results of this study, we propose that a large-scale study, including older adults’ health condition and its severity, be conducted to explore the use of physical restraints in nursing homes. In addition, it is necessary to compare the use of restraints between nursing homes with and without RNs to identify the impact of RNs and to bring in policy framework changes to improve restraint-related regulations.

## Data Availability

The datasets generated and/or analyzed during the current study are not publicly available (for protection of participants’ personal information) but are available from the corresponding author on request.
